# A Real-Time Mobile Robotic System for Crack Detection in Construction Using Two-Stage Deep Learning

**DOI:** 10.3390/s26020530

**Published:** 2026-01-13

**Authors:** Emmanuella Ogun, Yong Ann Voeurn, Doyun Lee

**Affiliations:** 1 Mechanical Engineering Department, Georgia Southern University, Statesboro, GA 30460, USA; eo05040@georgiasouthern.edu; 2 Civil Engineering and Construction Department, Georgia Southern University, Statesboro, GA 30460, USA; yv00447@georgiasouthern.edu

**Keywords:** crack detection, deep learning, U-Net, Pix2Pix, mobile robotics, autonomous inspection, structural health monitoring, semantic segmentation, ROS 2, SLAM

## Abstract

The deterioration of civil infrastructure poses a significant threat to public safety, yet conventional manual inspections remain subjective, labor-intensive, and constrained by accessibility. To address these challenges, this paper presents a real-time robotic inspection system that integrates deep learning perception and autonomous navigation. The proposed framework employs a two-stage neural network: a U-Net for initial segmentation followed by a Pix2Pix conditional generative adversarial network (GAN) that utilizes adversarial residual learning to refine boundary accuracy and suppress false positives. When deployed on an Unmanned Ground Vehicle (UGV) equipped with an RGB-D camera and LiDAR, this framework enables simultaneous automated crack detection and collision-free autonomous navigation. Evaluated on the CrackSeg9k dataset, the two-stage model achieved a mean Intersection over Union (mIoU) of 73.9 ± 0.6% and an F1-score of 76.4 ± 0.3%. Beyond benchmark testing, the robotic system was further validated through simulation, laboratory experiments, and real-world campus hallway tests, successfully detecting micro-cracks as narrow as 0.3 mm. Collectively, these results demonstrate the system’s potential for robust, autonomous, and field-deployable infrastructure inspection.

## 1. Introduction

### 1.1. Motivation and Problem Statement

Civil infrastructure, such as bridges, tunnels, and dams, is critical to public safety, economic stability, and national mobility. Due to constant use, these assets are continuously exposed to environmental influences, material fatigue, and aging, which can lead to progressive deterioration and, in extreme cases, catastrophic failure [1]. The consequences of such failures are often severe: historical incidents involving bridge collapses and dam breaches have resulted in loss of life, economic disruption, and long-term service interruptions [2].

Recent federal investments, particularly through the Infrastructure Investment and Jobs Act (IIJA), have contributed to measurable improvements in U.S. infrastructure conditions, as reflected in the 2025 American Society of Civil Engineers (ASCE) Infrastructure Report Card’s overall grade of C, an improvement from the C− in 2021 [3]. Despite this progress, critical vulnerabilities persist. Dams, for instance, received a D+, with over 15,400 classified as “high-hazard potential”, meaning their failure could result in fatalities [3]. Bridges present a similarly concerning picture: although they earned a C grade overall, approximately 7.5% remain structurally deficient, and the average age exceeds 46 years. Together, these conditions underscore the urgent need for proactive, frequent, and quantitative structural health monitoring (SHM).

Currently, visual inspection remains the most widely used initial assessment method due to its simplicity and non-destructive nature [4]. Inspections are typically performed at scheduled intervals or in response to observed distress, natural disasters, or planned retrofits. Manual inspection, conducted by trained human inspectors or with non-destructive testing (NDT) equipment, remains the most commonly used method of carrying out visual inspection. However, manual visual inspection suffers from well-documented limitations. Human inspectors face accessibility constraints in hazardous or confined spaces, are susceptible to fatigue and cognitive bias, and exhibit inter-observer variability [5]. This inherent subjectivity and inconsistency limit the reliability of manual inspection for critical infrastructure assessment.

Compounding these issues, high-risk assets are often under-inspected. A 2023 industry survey found that 68% of operators deferred or reduced inspection frequency for elevated or hazardous infrastructure due to safety concerns, operational downtime, and physical inaccessibility [6]. At the same time, the construction sector faces a severe labor shortage. The U.S. Bureau of Labor Statistics projects 663,500 annual job openings through 2033 [7], many of which remain unfilled due to the physically demanding nature of the work and elevated injury risk [8].

### 1.2. Sensor Technologies and Automated Inspection

In response to these challenges, researchers have explored automated inspection methods using advanced sensing technologies. Terrestrial laser scanning (TLS), for example, offers millimeter-level geometric accuracy but remains costly and time-intensive, thereby limiting its use for routine inspections [9]. In contrast, RGB and RGB-D imaging systems provide a more cost-effective alternative. However, traditional computer vision methods applied to these images, such as edge detection, thresholding, and morphological filtering, require extensive manual tuning and perform poorly under variable lighting, surface textures, or weather conditions [10].

Deep learning has significantly advanced visual inspection through automated crack detection, with architectures like U-Net and ResNet demonstrating strong performance on benchmark datasets [11]. Despite these advances, many early studies were evaluated on static, laboratory-grade images under controlled conditions [11], which restricts their real-world applicability. When deployed on mobile platforms, these models often struggle with domain shift, occlusions, and motion blur [10]. While recent work has improved robustness through domain adaptation, multi-sensor fusion, and lightweight models for edge deployment [12,13,14], few systems integrate real-time crack detection with autonomous navigation on a moving robotic platform in a field-deployable, end-to-end pipeline.

### 1.3. Research Scope and Contributions

Current approaches to automated infrastructure inspection face significant methodological limitations. Existing models often overlook the temporal consistency required during dynamic robot motion [15], or omit quantitative comparisons between controlled laboratory benchmarks and real-world field performance [10]. A detailed comparison of these existing methodologies against the proposed system has been performed and is described in Section 2.

This study focuses on visual crack detection as a first-stage screening tool within the broader structural health monitoring (SHM) ecosystem. While comprehensive structural damage assessment requires advanced techniques, such as modal frequency analysis [16], swarm intelligence algorithms [17], and deep learning on structural response data [18], visual inspection provides efficient large-area surveying to prioritize detailed analysis and document crack evolution over time. Detected cracks serve as inputs for subsequent engineering assessment.

Building on identified gaps in existing methodologies, this work presents a real-time, vision-based crack detection system integrated with autonomous mobile robot navigation. The scope of this work focuses on the fusion of deep learning-based visual inspection with LiDAR-based Simultaneous Localization and Mapping (SLAM). Specifically, the system utilizes 2D LiDAR data for mapping and autonomous navigation, while RGB data is processed exclusively for crack identification. It is important to note that while the broader robotic system hardware includes a manipulator, this paper concentrates on the integration of crack detection with autonomous mobility. Manipulator-based automated control is reserved for future research.

Through this integration of autonomous navigation and deep learning-based inspection, this work makes the following contributions:

1. Residual-Based Deep Learning Model: A two-stage model (U-Net + Pix2Pix) trained on segmentation residuals that enhances boundary precision for thin and branched cracks significantly.
2. Real-Time Edge Inference: A real-time detection pipeline optimized for edge deployment, evaluated on both onboard and distributed configurations to ensure consistent inference during autonomous navigation.
3. Autonomous Navigation Integration: The integration of a UGV navigation stack utilizing ROS 2 (vHumble), leveraging SLAM and adaptive Monte Carlo localization for consistent trajectory tracking.
4. Dynamic Evaluation: A comprehensive quantitative evaluation establishing robustness via mIoU, F1-score, precision, and recall, comparing static benchmarks against dynamic field performance.

## 2. Related Work

### 2.1. Visual Inspection and Structural Health Monitoring

Having established the limitations of manual inspection practices, this section examines the technical approaches researchers have developed to automate structural health monitoring (SHM). Early automation efforts focused on traditional image-processing techniques, such as thresholding and edge detection [19]. Although these methods improved consistency compared to manual evaluation, they relied on handcrafted feature engineering and lacked robustness. Their performance often degraded under challenging environmental conditions, including shadows, stains, and variable lighting, which led to significant drops in precision outside controlled conditions [20].

To improve coverage and safety, researchers shifted toward automated platforms, mounting sensors on robotic platforms [15]. For instance, in our previous research [21], we demonstrated that robotic arm operations could successfully automate complex construction tasks, validating the potential for robotics to replace manual labor in unstructured environments. However, the transition from static sensors to mobile robotics introduced new challenges in data fidelity. As we also reported in follow-up work, inherent limitations in image-based 3D reconstruction relying solely on camera poses can compromise inspection accuracy, particularly when depth information is not tightly integrated with visual data [22]. Consequently, current research has shifted toward deep learning-enabled mobile robotics to ensure both speed and scalability, aiming to close the gap between raw data collection and actionable real-time assessment [23].

**Figure 1:**
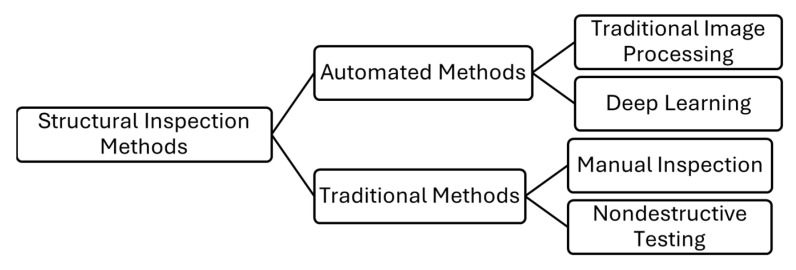
Structural Inspection Methods. The hierarchy distinguishes between traditional methods and automated methods.

This evolution, from manual inspection to image processing and finally to sensor-equipped robotics, is illustrated in Figure 1.

While recent efforts utilize automated platforms to capture data, they often rely on post-processing or manual review. Unlike these general data-collection platforms, the proposed work integrates the inspection logic directly onto the robot, enabling real-time, actionable detection rather than post-process analysis.

### 2.2. Deep Learning-Based Crack Detection

Due to the limitations of manual and traditional image-processing methods, recent research has focused on deep learning to automate crack detection [19,20]. These deep learning models offer superior feature extraction and robustness to environmental variations compared to prior methods [10]. Adapted originally from medical imaging [24], these architectures enable automated, consistent, and pixel-level inspection of civil infrastructure [25].

Within this field, three major approaches have emerged: image classification, object detection, and semantic segmentation [23]. Image classification assigns a binary label, such as “crack” or “no crack,” but lacks spatial localization [26]. Object detection addresses this limitation by using bounding boxes [23]. Two-stage detectors like Faster R-CNN offer high precision [27], while single-stage detectors like the YOLO series are preferred for real-time efficiency [28]. Although recent iterations, such as YOLOv5s, provide strong accuracy [29], bounding-box predictions fail to capture irregular crack geometries or complex branching patterns.

**Figure 2:**
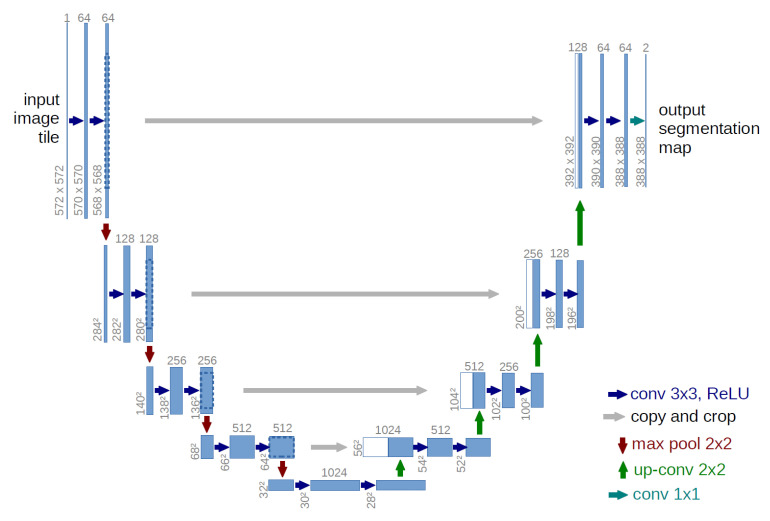
Schematic representation of the U-Net architecture adopted for pixel-wise crack segmentation [24].

To achieve detailed crack mapping, semantic segmentation performs pixel-level classification [30]. Architectures like U-Net, SegNet, and DeepLab are dominant [26,27]. Among these, U-Net is preferred for its balance of accuracy and efficiency, particularly with limited datasets [31]. Its encoder–decoder structure with skip connections preserves spatial information critical for precise localization [24], as illustrated in Figure 2.

Despite its strengths, U-Net often produces blurred boundaries for thin cracks and generates false positives in textured or shadowy environments [29,30]. As identified by our previous study [32], high-precision pixel tracking is essential for effective robotic automation, yet standard segmentation models often fail to maintain this fidelity under dynamic conditions. To overcome these challenges, researchers have explored Generative Adversarial Networks (GANs) for image enhancement and super-resolution [33,34]. Methods like SRCNet [35] and RP-GAN [36] enhance crack image detail by increasing resolution and sharpening features, but full-image super-resolution requires significant computational overhead. Residual refinement offers a more practical alternative by applying generative processing only to regions requiring correction, making it suitable for deployment on edge devices.

Overall, existing approaches force a trade-off: high-speed detection with low precision (YOLO) or high-precision segmentation (standard U-Net) that suffers from boundary blurring. This study addresses this trade-off by introducing a hybrid two-stage approach that combines a residual-based U-Net for initial segmentation with a Pix2Pix GAN for targeted refinement. Together, these components create a pipeline that is both accurate and computationally optimized for real-time edge inference.

### 2.3. Integrated Autonomous Robotic Inspection Systems

Autonomous robotic inspection has evolved from isolated hardware experiments into integrated systems unifying Platform, Cognition, and Action [37]. This framework addresses challenges in GPS-denied environments and the need for spatially registered maps [38]. While UAVs provide rapid coverage of elevated structures [39,40] and climbing robots excel in vertical or confined spaces [41], UGVs are optimal for close-range, high-precision inspection in confined or GPS-denied environments [36,37]. The proposed system utilizes a UGV with a streamlined sensor suite (e.g., RealSense RGB-D and 2D LiDAR) to balance fidelity with industrial cost-effectiveness [42].

Additionally, real-time inference is crucial for adaptive inspection [43]. Recent work in vision-based construction robots emphasizes that real-time interaction capabilities are critical for effective human–robot collaboration [44]. Furthermore, studies on joint tracking have shown that automatic, real-time scanning is a prerequisite for high-fidelity robotic tasks [32]. Consistent with these findings, the proposed system implements a distributed ROS 2 architecture. It partitions tasks: an onboard computer handles safety-critical navigation, while an offboard GPU manages the two-stage AI pipeline to ensure responsiveness. Reliable localization is vital in GPS-denied sites. While Vision-based SLAM is common, it falters under poor lighting. Recent research validates that LiDAR-centric navigation (e.g., Cartographer, AMCL) provides the necessary robustness for autonomous positioning in mobile robotic applications [45]. The proposed system leverages this validated approach, using the SLAM toolbox for mapping and Adaptive Monte Carlo Localization (AMCL) for precise localization [46].

Many existing systems prioritize isolated components, either platform mechanics or offline algorithms. However, this work is distinct because it unifies these elements. It combines robust LiDAR-based SLAM (avoiding the pitfalls of Visual SLAM) with a real-time generative AI model, providing a complete “scan-and-assess” solution that previous single-domain studies lack.

## 3. System Architecture and Hardware Platform

This section outlines the system architecture of the proposed approach and provides detailed descriptions of the hardware and software platforms used in the experimental setup.

### 3.1. System Overview

**Figure 3:**
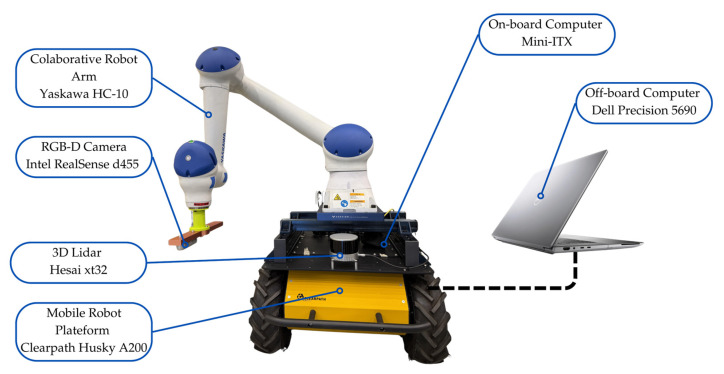
Hardware Architecture of the Mobile Perception Platform, showing the UGV base, the robotic arm, and the offboard computer handling advanced processing.

The proposed two-stage real-time crack detection system is built upon a tightly integrated hardware and software architecture designed for autonomous infrastructure inspection. As illustrated in Figure 3, the system architecture comprises three main layers: (1) the mobile robotic platform equipped with multimodal sensors, (2) an onboard computing unit running a modular software stack, and (3) a two-stage deep learning pipeline for crack detection and refinement. This layered design enables robust perception, autonomous navigation, and high-fidelity defect identification in real-world environments. The robotic arm (see Figure 3) is a component for future work in crack profiling, not currently used for real-time crack detection, but intended for closer inspection and quantifying crack characteristics.

#### 3.1.1. Hardware Components

The mobile platform is a Clearpath Husky UGV A200 (Kitchener, ON, Canada), selected for its robustness and all-terrain capability. It is equipped with two primary sensors:

1. A Hesai Technology (Shanghai, China) PandarXT-32 LiDAR (see Figure 3), featuring a 360° horizontal field of view (FOV), a 31° vertical FOV (−16° to +15°), 32 laser channels, and a detection range of 0.05 m to 120 m. It delivers up to 640,000 points/second in single-return mode and 1,280,000 points/second in dual-return mode, with an IP6K7 ingress protection rating for outdoor durability.
2. An Intel (Santa Clara, CA, USA) RealSense D455 RGB-D camera (see Figure 3), providing synchronized high-resolution color and depth streams up to 90 fps.

Computation is distributed across two systems:

1. The onboard computer is a Mini-ITX single-board system (Intel i3-9100TE quad-core CPU, 16 GB DDR4 RAM, 250 GB SSD) with Ethernet, USB 3.0, RS232, and PCIe 3.0 × 16 expansion. It runs a minimal ROS 2 Humble instance and is responsible for low-level robot control, sensor data acquisition, and real-time communication with the offboard unit.
2. The offboard computer is a Dell (Round Rock, TX, USA) Precision 5690 mobile workstation powered by an Intel Core Ultra 7 processor and an NVIDIA (Santa Clara, CA, USA) RTX 2000 Ada Generation GPU. This unit handles computationally intensive tasks, including SLAM, path planning, and the two-stage AI crack detection pipeline.

The software framework is built on ROS 2 Humble, leveraging its real-time communication capabilities and modular node architecture across the two machines via a high-bandwidth Ethernet link. Key components include:

- Mapping and Localization: The SLAM toolbox performs LiDAR-based SLAM to generate a 2D occupancy grid map. Once mapped, AMCL localizes the robot within the map, and the NAV2 stack manages autonomous navigation with obstacle avoidance.
- AI Inference Pipeline: This pipeline implements a two-stage deep learning approach: (1) a U-Net model performs pixel-wise semantic segmentation to identify potential cracks in RealSense RGB-D frames, and (2) a Pix2Pix conditional GAN refines these predictions by enhancing crack continuity and suppressing noise or false positives. Both models are optimized for real-time inference using TensorRT.

#### 3.1.2. System Architecture and Data Flow

The inspection process follows a structured sequence:

1. Upon deployment, the robot begins streaming LiDAR and RGB-D data to the offboard workstation.
2. The Slam Toolbox incrementally builds a global map of the environment, which is visualized in real-time.
3. An operator defines a region of interest (ROI) for inspection via an intuitive graphical interface overlaid on the map.
4. NAV2 computes and executes a safe trajectory to the ROI, with the onboard computer managing motor control and obstacle response. As the robot navigates the path, the RGB-D camera continuously captures surface imagery.
5. Simultaneously, the offboard system processes each frame through the U-Net + Pix2Pix pipeline, producing refined crack detections that are logged, visualized, and made available for downstream decision-making.

**Figure 4:**
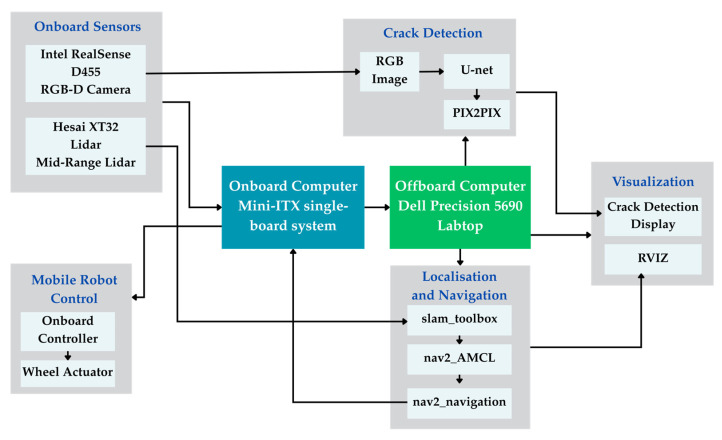
System architecture for autonomous crack inspection. The schematic illustrates the connection between onboard sensors, the mobile robot controller, and the dual-computer processing setup. Key software modules for Crack Detection (U-net, Pix2Pix) and Navigation (Nav2, AMCL) are executed on the offboard computer to enable autonomous inspection and visualization.

By decoupling control and perception workloads, this architecture achieves a practical balance between autonomy, computational efficiency, and inspection accuracy, making it well-suited for real-world deployment in civil infrastructure monitoring scenarios. This sequence is illustrated in Figure 4.

## 4. Methodology

This section details the methodology adopted for developing and evaluating the proposed real-time crack detection system. It includes the dataset preparation process, model architecture, and training configuration, as well as the evaluation metrics and experimental procedures used to assess performance.

### 4.1. Dataset Preparation and Model Training

#### 4.1.1. Dataset Description

The proposed system was trained and evaluated using the CrackSeg9k dataset, a comprehensive collection of 9255 annotated crack images curated from multiple infrastructure types, including pavements, bridges, buildings, and tunnels [47]. The images were gotten from multiple existing crack datasets, and each annotation was manually verified to ensure label quality. Every image is paired with a corresponding binary mask in which crack pixels are labeled as foreground (value 1) and non-crack regions as background (value 0) [47]. These ground truth masks served as the supervisory signal for training both the U-Net baseline model and the Pix2Pix refinement network.

**Figure 5:**
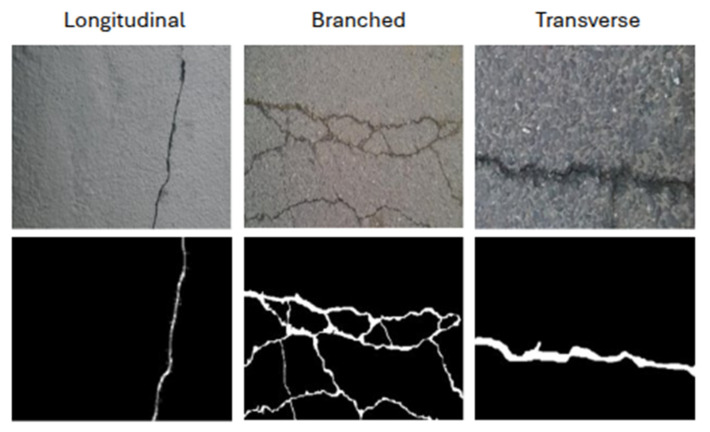
Samples from CrackSeg9k showing various crack patterns and corresponding annotations.

The images in the dataset exhibit significant variability in terms of crack morphology, surface texture, lighting conditions, and viewpoint angles, making it well-suited for developing robust crack detection models that can generalize to real-world inspection scenarios. Crack types represented in the dataset include longitudinal, transverse, branched, and block cracking patterns, with varying widths ranging from hairline cracks (<1 mm) to severe structural cracks (>5 mm), as illustrated in Figure 5.

For model development, the dataset was split into training (70%), validation (15%), and test sets (15%) using stratified random splitting with a fixed seed to ensure reproducibility.

#### 4.1.2. Data Preprocessing and Augmentation

To standardize input dimensions and facilitate efficient batch processing, all images were resized to 384 × 384 pixels during training and validation. This resolution was selected to balance computational efficiency with the preservation of fine crack details, particularly important for detecting narrow cracks. The Albumentations library (v2.0.8), a fast and flexible library for image augmentation [48], was employed to implement the data preprocessing pipeline.

Data augmentation plays a critical role in improving model generalization and robustness to variations in real-world imaging conditions. The augmentation pipeline was applied on the fly and included geometric transformations, photometric adjustments, and image degradation effects. These operations increased the diversity of the training data and helped the model remain robust to changes in viewpoint, lighting, and imaging noise. The set of transformations used in this work is listed in Table 1.

All stochastic augmentation techniques were applied exclusively to the training set. Validation and testing data were limited to resizing and normalization.

### 4.2. Model Architecture and Training Setup

**Figure 6:**
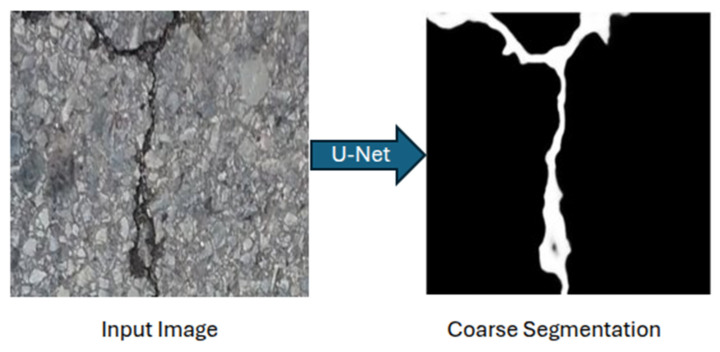
U-net segmentation on the input image. The model processes the raw input image (**left**) to generate a binary coarse segmentation mask (**right**) that highlights the crack structure.

**Figure 7:**
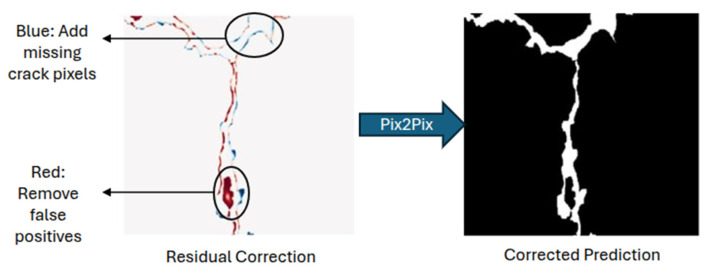
Visualization of the residual refinement process. The U-Net produces an initial segmentation, which is refined by learning pixel-wise corrections. Blue regions indicate missed crack pixels (false negatives), red regions indicate false positives to be removed, and gray regions represent correctly classified areas. The color overlay is used for visualization; the corresponding residuals are encoded as grayscale images during Pix2Pix training.

The proposed system combines a U-Net for initial segmentation with Pix2Pix for residual refinement. The U-Net generates coarse crack predictions (Figure 6), while Pix2Pix learns to correct systematic errors such as boundary blur, fragmentation, and false positives, through adversarial training on residual maps (Figure 7). This setup enables high accuracy while maintaining real-time processing capability.

#### 4.2.1. U-Net Training Configuration

The U-net model accepts 3-channel RGB images (384 × 384 × 3) as input and produces single-channel binary segmentation masks (384 × 384 × 1) as output. A combined loss function was employed to leverage the complementary strengths of pixel-wise and region-based optimization objectives. The loss function is defined as:

(1) $$L_{combined}=0.6\cdot L_{BCE}+0.4\cdot L_{Dice}\;$$

Binary Cross-Entropy (BCE) loss handles pixel-wise classification and penalizes misclassifications at the individual pixel level, ensuring spatial accuracy. On the other hand, Dice loss addresses class imbalance inherent in crack detection (where crack pixels typically constitute less than 10% of the image) by directly optimizing the overlap between predicted and ground truth regions. The weighting ratio (60% BCE, 40% Dice) was determined through preliminary experiments and provides stable training dynamics while maintaining sensitivity to fine cracks.

The model was trained using the AdamW optimizer with an initial learning rate of 1 × 10^−4^ and weight decay of 1 × 10^−4^ to prevent overfitting. A ReduceLROnPlateau scheduler monitored validation loss and reduced the learning rate by a factor of 0.5 when no improvement was observed for 10 consecutive epochs, enabling the model to escape local minima and achieve finer convergence. In addition, training was conducted for 100 epochs with a batch size of 8. Mixed precision training (FP16) was employed using PyTorch (v2.5.1) automatic mixed precision (AMP) to reduce memory consumption and accelerate training without sacrificing model performance. Each training epoch required approximately 5 min, resulting in a total training time of approximately 8.5 h per model. To ensure statistical reliability, three independent training runs were conducted with different random seeds (42, 123, 456), and results are reported as mean ± standard deviation across these trials.

The best model checkpoint was selected based on the highest validation F1 score, and these checkpoints were saved every 10 epochs to enable analysis of training dynamics and facilitate recovery from training interruptions.

#### 4.2.2. Pix2Pix Refinement Training

The Pix2Pix conditional GAN was trained as a second-stage refinement module after the U-Net had converged. Instead of generating a new segmentation mask, the Pix2Pix generator learns the residual correction needed to transform the U-Net’s prediction into the ground truth. This focuses refinement on specific errors such as boundary blur, missing crack segments, and false positives. Given a U-Net prediction $M_{U-Net}$ and ground truth mask $M_{GT}$, the residual R is computed as:

(2) $$R=M_{GT}-M_{U-Net}$$

To generate training pairs, the trained U-Net model performed inference on all training images, producing binary prediction masks. The residual maps were then computed by subtracting these U-Net predictions from the ground truth masks. Since both the U-Net outputs and ground-truth annotations are binary, the resulting residual map *R* takes values in the discrete set {−1, 0, +1}, where

- *R* = +1 denotes false negatives (crack pixels missed by the U-Net);
- *R* = 0 denotes correct predictions (either crack or background);
- *R* = −1 denotes false positives (non-crack pixels incorrectly classified as cracks).

For Pix2Pix training, the residual maps were converted into 8-bit grayscale images using a linear mapping:

- +1 is mapped to 255 (white; pixels to be added),
- 0 is mapped to 127 (gray; no correction required)
−1 is mapped to 0 (black; pixels to be removed).

This encoding enables the Pix2Pix generator to learn spatially coherent correction patterns, allowing it to refine crack boundaries and suppress false positives rather than re-predicting the full segmentation.

Both the U-Net predictions (input) and residual maps (target) were saved as grayscale images and organized into separate directories for efficient data loading during the Pix2Pix training process. Furthermore, the Pix2Pix framework consists of a U-Net-style generator that learns the residual transformation and a PatchGAN discriminator that classifies local image patches as real or generated.

The training objective combines adversarial loss and L1 reconstruction loss:

(3) $$L_{Pix2Pix}=\;L_{GAN}+\lambda \cdot L_{L1}$$

where $L_{GAN}$ is the adversarial loss that encourages realistic crack textures and edge sharpness, $L_{L1}$ is the L1 distance between predicted and target residuals that preserves spatial accuracy.

The models were trained for 100 epochs with a batch size of 8, using the Adam optimizer with a learning rate of 2 × 10^−4^ and beta coefficients (0.5, 0.999), with the generator and discriminator being updated alternately in each training iteration. The discriminator was trained first on both real and fake pairs, followed by generator training with the discriminator’s gradients frozen. Mixed precision training was again employed to manage GPU memory constraints.

### 4.3. Performance Metrics and Evaluation

Model performance was evaluated using standard semantic segmentation metrics that assess both pixel-level accuracy and region-level overlap:

- Intersection over Union (*IoU*): Measures the overlap between predicted and ground truth crack regions, computed as

(4) $$IoU=\frac{TP}{TP+FP+FN}$$

where TP, FP, and FN represent true positives, false positives, and false negatives at the pixel level.

- Mean IoU (*mIoU*): The average *IoU* across both classes (crack and background), providing a balanced metric for binary segmentation:

(5) $$mIoU=\frac{1}{2}\left({IoU}_{crack}+{IoU}_{background}\right)$$

- *Precision*: The ratio of correctly predicted crack pixels to all predicted crack pixels, quantifying the model’s ability to avoid false positives:

(6) $$Precision=\frac{TP}{TP+FP}$$

- Recall: The ratio of correctly predicted crack pixels to all actual crack pixels, measuring the model’s sensitivity to crack presence:

(7) $$Recall=\frac{TP}{TP+FN}$$

- F1 Score: The harmonic mean of precision and recall, providing a single metric that balances both:

(8) $$F1=2\cdot \frac{Precision\cdot Recall}{Precision+Recall}$$

### 4.4. Robot Software Integration and Operational Workflow

The trained U-Net + Pix2Pix model was deployed within a ROS 2 Humble framework to enable real-time crack detection during autonomous mobile robot operations. The system was implemented as a modular perception node that interfaces seamlessly with standard robotic navigation and localization components, ensuring compatibility with existing UGV platforms and facilitating future deployment on different robotic systems.

**Figure 8:**
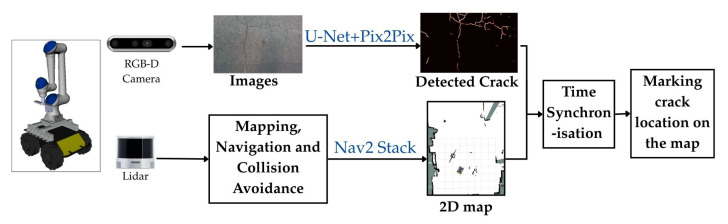
ROS 2-based Integration of Perception, Localization, and Visualization Modules. This modular design uses separate threads for perception (U-Net/Pix2Pix) and navigation (Nav2 Stack), with time synchronization to fuse crack detections onto the global 2D map in real-time.

The crack detection node subscribes to RGB and depth image streams from the camera mounted on the mobile platform, processing each frame through the proposed algorithm pipeline. Detection outputs, including segmentation masks and confidence metrics, are published for navigation, planning, and logging via modular ROS 2 topics, as shown in Figure 8. Spatial localization of detected cracks is achieved through integration with the AMCL package, which provides continuous pose estimates in the global map frame.

Visualization was implemented using RViz markers, where persistent red spherical indicators are placed at the robot’s position whenever cracks are detected, creating a cumulative spatial map of all identified cracks over the inspection mission. This modular design allows for straightforward adaptation to different camera configurations, alternative localization systems, or enhanced downstream processing modules without requiring modifications to the core detection pipeline. Processing occurs entirely offboard computer, ensuring robust operation in environments with limited network connectivity.

#### 4.4.1. Mapping and Localization

The system uses the 3D LiDAR in processing a single horizontal scan ring to generate 2D laser scans for computational efficiency. During the mapping phase, SLAM Toolbox builds a globally consistent 2D occupancy grid map in real time by fusing this 2D LiDAR data with wheel odometry and IMU as motion priors. Leveraging scan matching and loop closure within a pose-graph framework, SLAM Toolbox produces a metrically accurate map suitable for long-term use. Once the map is generated and saved, the system switches to localization mode using AMCL, which provides robust and efficient pose estimation against the pre-built map. This two-stage pipeline, SLAM for mapping and AMCL for localization, is a well-established practice in ROS-based navigation, offering tuning flexibility and runtime stability.

#### 4.4.2. Navigation, Collision Avoidance, and Path Planning

Navigation is managed via the ROS 2 NAV 2 stack, which orchestrates global and local planning to achieve safe, goal-directed motion. The global planner computes collision-free paths through the occupancy grid toward operator-specified regions of interest. Concurrently, the local planner, configured with a Dynamic Window Approach (DWA), continuously generates short-horizon, dynamically feasible trajectories that avoid both static obstacles (e.g., barriers, curbs) and dynamic hazards (e.g., construction personnel or equipment), using real-time LiDAR data. To ensure high-quality visual data capture during inspection, Husky’s low-level controller adapts its velocity based on terrain roughness, inferred from IMU accelerations or LiDAR point cloud density.

#### 4.4.3. Multi-Sensor Fusion and Real-Time Coordination

Temporal and spatial alignment of heterogeneous sensor streams is achieved through software-based time synchronization using ROS 2’s message_filters::ApproximateTimeSynchronizer, which aligns LiDAR, RGB-D, and odometry messages within a configurable tolerance window. To accommodate the bandwidth constraints of the onboard-offboard Ethernet link, a dedicated watchdog node continuously monitors system health, tracking metrics including sensor data liveness, GPU memory utilization, CPU load, and network round-trip time. If a critical anomaly is detected, such as LiDAR dropout, GPU inference stall, or communication timeout, the watchdog initiates fail-safe protocols, which may include immediate motion halt or transition to manual mode. This layered robustness ensures reliable operation in real-world infrastructure environments where sensor occlusions, wireless interference, or computational bottlenecks are common.

#### 4.4.4. Operational Workflow

**Figure 9:**
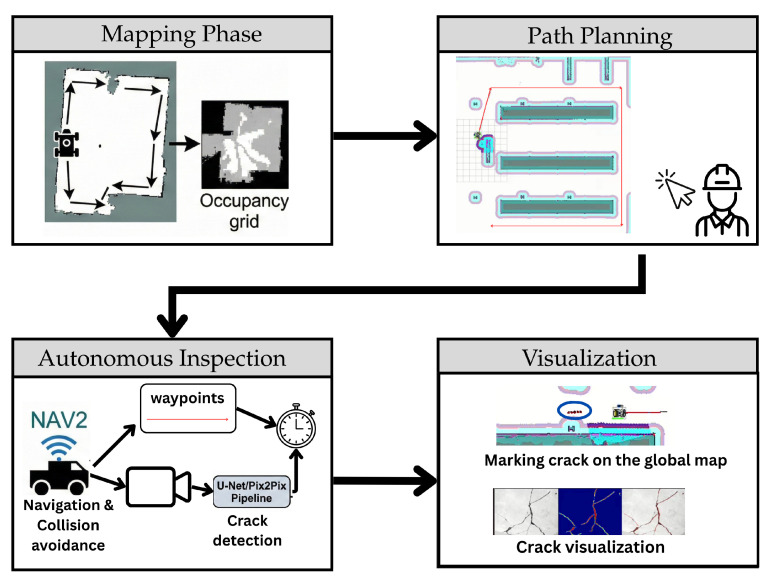
System Workflow. The four-stage pipeline consists of manual mapping, path planning, autonomous inspection (via NAV2 and U-Net/Pix2Pix), and defect visualization.

The complete inspection mission follows a structured, four-stage workflow as illustrated in Figure 9:

1. Mapping Phase: The operator manually drives the robot to explore the environment. This manual traversal enables the system to construct a static occupancy grid, distinguishing between free space and obstacles to serve as the foundation for future navigation.
2. Path Planning: an operator interacts with the system to define the mission. Using the generated map, the operator manually selects regions of interest or inspection zones, establishing the trajectory waypoints the robot will follow.
3. Autonomous Inspection: The execution phase relies on NAV2 for robust navigation and collision avoidance. The robot autonomously traverses a sequence of predefined waypoints to reach inspection targets. Simultaneously, the onboard camera captures high-resolution imagery, which is streamed to an offboard computer for real-time processing through a U-Net/Pix2Pix pipeline, enabling immediate crack detection without overburdening the robot’s onboard computer resources.
4. Visualization: The final stage focuses on data presentation. Detected defects are spatially registered, marking cracks on the global map for localization. Additionally, the system generates detailed crack visualization, displaying the raw imagery alongside the segmented crack detection results to facilitate assessment.

## 5. Experimental Validation

The proposed AI-based autonomous crack detection robotic system was validated through a tiered experimental framework spanning simulation, controlled laboratory trials, and real-world indoor deployments. This approach was designed to evaluate three critical dimensions of system performance: (1) robust autonomous navigation and mapping in construction-representative environments, (2) accuracy and generalization of crack detection across varying substrates, orientations, and visual artifacts (including photorealistic decoys), and (3) end-to-end computational efficiency and operational stability under realistic deployment conditions.

### 5.1. Simulation and System Integration

Initial system integration and algorithm tuning were performed in Gazebo Fortress LTS (Open Robotics, San Jose, CA, USA), leveraging its ROS 2-native physics simulation capabilities. A custom virtual environment was constructed to mimic a concrete floor, featuring a textured concrete surface with synthetically embedded cracks. These cracks were modeled using high-resolution displacement maps and physically based rendering to simulate realistic lighting, shadowing, and surface degradation. The robot was tasked with autonomously navigating and mapping a 30 m × 40 m arena while the perception pipeline processed simulated RGB-D streams in real-time to detect surface anomalies.

**Figure 10:**
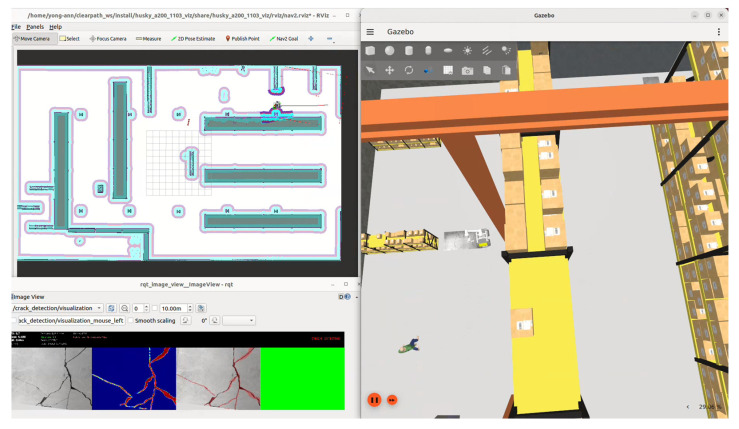
Simulation Visualization of Autonomous Crack Inspection. The setup shows the Gazebo environment (**right**) and the RViz map (**top left**), where real-time crack detections are back-projected onto the global map as red markers, validating the system’s ability to localize and map surface anomalies. (**Bottom Left**) The raw output from the U-Net + Pix2Pix crack detection pipeline (rqt_image_view).

During autonomous traversal, the crack detection pipeline processed each incoming RGB frame to generate pixel-level crack masks. Using precise hardware-level time synchronization between the simulated RGB-D camera and the robot’s odometry, the detected crack locations were back-projected into the global map frame. The detections were then visualized in real-time as red color markers overlaid on the Cartographer-generated occupancy grid within RViz, enabling immediate spatial validation of detection accuracy and localization. The visualization of this setup is seen in Figure 10.

This stage confirmed successful synchronization between the LiDAR and camera streams, validated Cartographer’s convergence on repetitive concrete textures, and demonstrated that the two-stage crack-detection model could generalize to synthetic defects without overfitting to unrealistic patterns. Critically, the end-to-end simulation pipeline, including navigation, perception, temporal alignment, and map annotation, proved robust and responsive, allowing rapid iteration of both navigation parameters (e.g., planner tolerances, inflation radii) and perception thresholds (e.g., segmentation confidence, post-processing filters) prior to hardware deployment. This significantly reduced field debugging time and increased confidence in the system’s readiness for physical testing. The recorded video of the Gazebo simulation demonstrating the autonomous crack detection system can be found at https://youtu.be/TNtV2WLi4f8 [49].

### 5.2. Physical Environment Evaluation

Following successful simulation validation, the system was rigorously evaluated in physical environments spanning controlled laboratory conditions and complex, operational indoor spaces. These trials were designed to assess detection fidelity, navigation robustness, and system resilience under realistic inspection scenarios.

#### 5.2.1. Laboratory Testing

**Figure 11:**
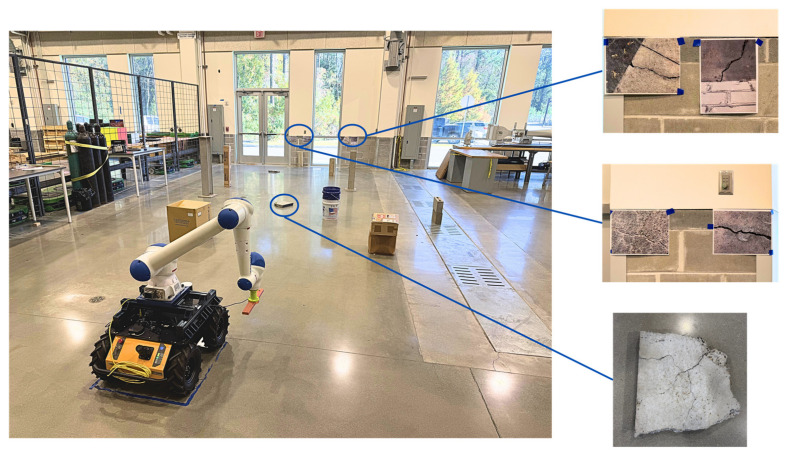
Laboratory Testing Environment used to evaluate the autonomous mobile robotic system’s performance against multiple crack modalities, including floor cracks, wall-mounted cracks, and photorealistic decoys.

In the laboratory setting, three distinct crack modalities were tested to evaluate the perception system’s versatility and robustness:

(a) Floor crack scenario: A concrete slab with a surface crack was placed on the floor, as shown in Figure 11. The robot autonomously navigated to the regions of interest, and the camera captured high-fidelity RGB data. The recorded video of the laboratory floor experiment can be found at https://youtu.be/tSlldbQ8O78 [50].
(b) Wall-mounted cracks: A vertical concrete slab with a surface crack was mounted on a wall to emulate bridge or retaining wall inspection contexts. The robot adjusted the arm-mounted camera to maintain near-perpendicular viewing geometry, ensuring consistent image scale and minimal perspective distortion.
(c) Photorealistic 2D validation: To validate the model’s visual pattern recognition capabilities, high-resolution printed images of cracks were affixed to a wall. These images matched real defects in morphology and color but lacked physical depth.

This setup confirmed that the algorithm could successfully generalize to crack morphologies and textures in a controlled 2D environment before introducing depth-based complexity. The recorded video of the laboratory wall experiment, demonstrating the inspection of both printed images and actual crack samples, can be foundat https://youtu.be/2fDa-pLnY50 [51].

**Figure 12:**
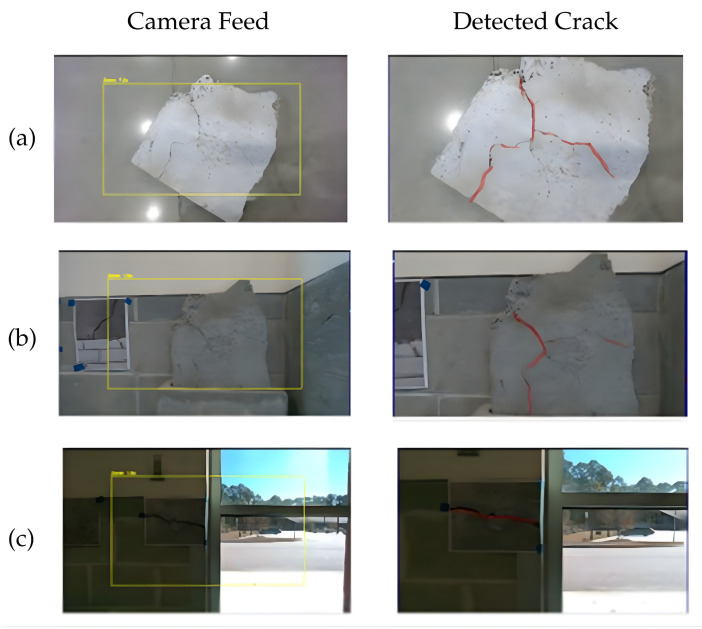
Performance of the perception system across different test setups. (**a**) Floor crack scenario; (**b**) Wall-mounted crack scenario; (**c**) a photorealistic printed crack decoy. The red overlay indicates the segmentation output generated by the deep learning model.

The system correctly detected all cracks in scenarios (a) and (b), and the printed decoys in (c) in Figure 12, demonstrating the effectiveness of the two-stage deep learning pipeline (U-Net + Pix2Pix).

#### 5.2.2. Real-World Indoor Deployment

**Figure 13:**
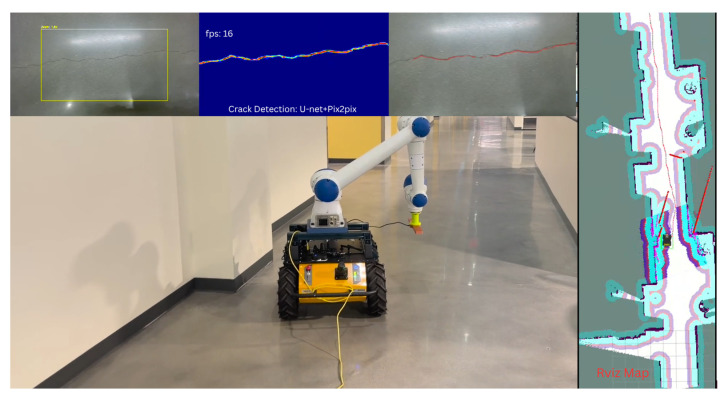
Real-Time Visualization of the Complete Autonomous Crack Mapping Pipeline in an Unstructured Hallway. The visualization includes the RViz Map (**Right**), showing the Cartographer-generated occupancy grid with the robot’s dynamically replanned, collision-free trajectory (NAV2 stack). The real-time crack detection output (**Top Left Insets**), displaying the RGB frame, the U-Net + Pix2Pix-generated segmentation mask, and the corresponding confidence metrics. The system maintained real-time operation, achieving approximately 16 fps for the detection pipeline.

For system integration and controlled validation, the system was deployed in an indoor walkway within a university campus building. The 30 m-long corridor featured heterogeneous flooring (including polished concrete with spalling, tile transitions, and highly reflective surfaces), dynamic lighting with overhead LED glare. This environment presented realistic challenges such as specular reflections, repetitive textures, and transient occlusions. The robot operated during off-peak hours under full autonomy. Users defined inspection ROIs via RViz on the live Cartographer-generated occupancy map (0.05 m resolution). The NAV2 stack generated dynamically replanned, collision-free trajectories (see Figure 13).

Across five consecutive runs, the system maintained stable localization and successfully identified naturally occurring micro-cracks (0.3–1.2 mm wide) in the concrete flooring. The recorded video of the real-world deployment on the GSU campus walkway can be found at https://youtu.be/S_Qddbrxurk [52].

#### 5.2.3. Visual Robustness Evaluation Using Real-World Images

To further assess the robustness of the proposed pipeline under unrefined real-world visual conditions, additional experiments were conducted using RGB images captured with the Intel RealSense camera. A total of 80 images were collected around the university campus, exhibiting challenging visual artifacts commonly encountered during practical inspections, including non-uniform illumination, strong shadows, surface reflectance, and background texture variability.

These images were not included in training, and no additional fine-tuning was performed, allowing the assessment to focus solely on the generalization capability of the trained model. Across the evaluated images, the proposed U-Net + Pix2Pix pipeline successfully detected cracks in 76 out of 80 cases, with confidence values ranging between 0.6 and 0.8. Missed detections were primarily associated with extremely low-contrast cracks under heavy shadowing, where the crack-to-background intensity difference was minimal.

**Figure 14:**
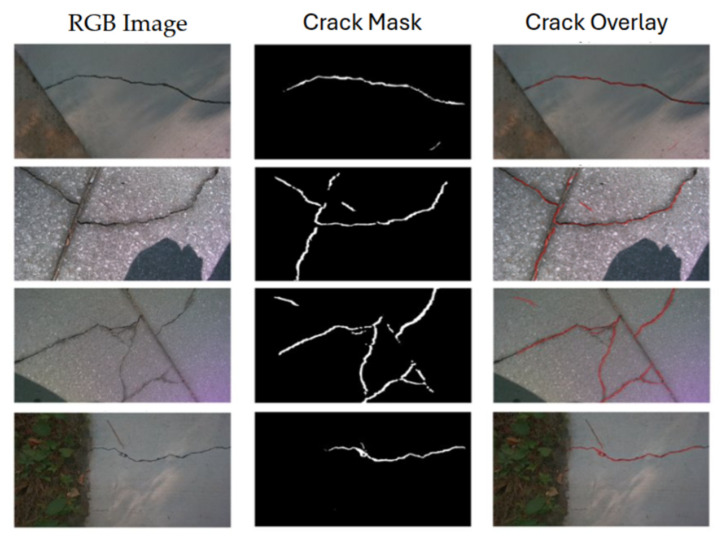
Real-world crack detection under varied visual conditions. Results from campus infrastructure testing showing RGB images captured with RealSense D455 (**left**), generated binary crack masks (**center**), and crack overlay visualizations (**right**). The system maintains detection accuracy across diverse illumination scenarios, demonstrating practical robustness for field deployment.

The qualitative results, including the original RGB images, the generated crack masks, and the final crack overlays, are presented in Figure 14. Navigation robustness in unstructured outdoor construction environments has been validated in our prior work [45], which demonstrated stable SLAM and collision avoidance on the same UGV platform.

### 5.3. Quantitative Evaluation

#### 5.3.1. Static Dataset Analysis

The evaluation metrics were computed on the test set after training completion, based on the results obtained in our previous research [53]. Additionally, inference speed was measured in frames per second (fps) on the target hardware to assess real-time processing capabilities. To ensure a consistent and rigorous performance baseline, all models listed in Table 2 were evaluated using the CrackSeg9k dataset.

The YOLO-based segmentation models were trained using the same dataset splits, data augmentation strategies, and training protocol as the U-Net baseline to ensure a fair comparison. The nano variants were selected to satisfy real-time processing constraints required for robotic deployment [14].

The relatively lower mIoU values obtained by the YOLO models (26.3–29.5%) can be attributed to several factors. First, YOLO architectures are primarily designed for object detection and bounding-box regression [28], which makes precise pixel-level segmentation of thin and irregular crack structures more challenging compared to fully convolutional segmentation networks [57]. Second, the nano variants prioritize computational efficiency and inference speed, resulting in reduced feature representation capacity for fine-grained crack patterns [14].

In addition, the CrackSeg9k dataset presents substantial challenges due to its severe class imbalance, complex crack morphologies, and varying illumination and background conditions [30,44]. These characteristics disproportionately affect detection-based models, whereas encoder–decoder architectures such as U-Net are better suited for dense pixel-wise prediction tasks [10].

As shown in Table 2, the proposed two-stage approach achieves 73.9% mIoU on CrackSeg9k, positioning it between the U-Net baseline (70.2%) and specialized lightweight segmentation models such as HrSegNet-B48 (80.3%) and CrackScopeNet (82.1%). While these specialized architectures demonstrate superior accuracy through crack-specific design optimizations, the proposed residual refinement approach offers competitive performance with a modular architecture that enables flexible deployment, using the U-Net alone when computational constraints are critical, or the full refinement pipeline when improved accuracy justifies the additional overhead. Overall, the results demonstrate that the proposed residual learning framework effectively enhances segmentation accuracy while preserving real-time performance.

#### 5.3.2. Computational Cost Analysis

The two-stage architecture introduces measurable computational overhead beyond the baseline U-Net model. The resource requirements for residual refinement are quantified in Table 3.

The proposed framework increases total parameters by 218% and FLOPs by 56%. This architectural expansion is the primary driver for the increase in static inference latency from 22.1 ms to 26.4 ms. However, this trade-off is justified by a 3.7% improvement in mIoU, reaching 73.9% (Table 2). This gain is particularly significant for the detection of hairline cracks and the suppression of texture-induced false positives, which the baseline model often struggles to resolve. Despite the +19% increase in inference time, the system maintains a throughput of 37.9 fps (Table 2) in static testing, comfortably exceeding the requirements for real-time robotic integration.

#### 5.3.3. Real-Time System Performance

The system-level metrics during real-world robotic deployment are summarized in Table 4. The refined segmentation pipeline achieved 16 fps with 62.5 ms end-to-end latency on the offboard GPU configuration, satisfying real-time constraints (>10 fps minimum). The temporal filter (4/5 frame persistence) effectively balanced false positive suppression and crack detection completeness: lowering to 3/5 increased false detections by 27%, while raising to 5/5 missed 15% of genuine cracks during rapid motion.

### 5.4. Qualitative Analysis and Real-Time Detection

**Figure 15:**
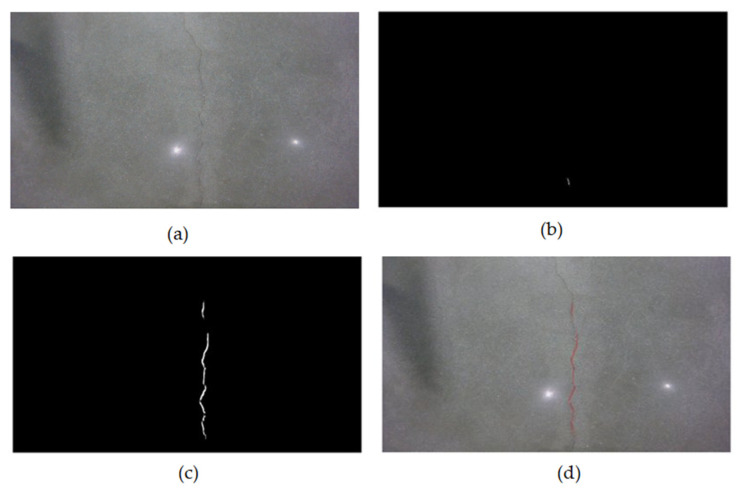
Detection comparison of crack segmentation using the U-Net baseline and Pix2Pix refinement. (**a**) input image, (**b**) U-Net output, (**c**) Pix2Pix refinement, (**d**) final overlay. The Pix2Pix refinement stage sharpens boundaries, reduces false positives on textured backgrounds, and improves crack continuity, especially in challenging low-contrast scenarios.

The detection results comparing the U-Net baseline and Pix2Pix refinement outputs are shown in Figure 15. The refined segmentation approach demonstrates improved boundary continuity, reduced false positives on textured backgrounds, and recovery of fragmented crack segments, particularly evident in challenging scenarios with shadows and low-contrast cracks.

**Figure 16:**
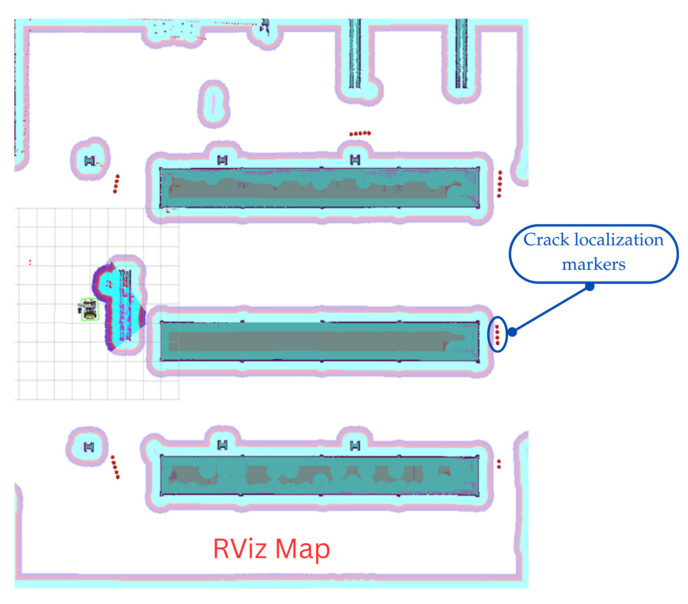
Map Visualization Showing Crack Localization. The red dot markers indicate the locations of structural defects across the inspection area. The RViz visualization confirms the successful temporal synchronization between the crack detection pipeline and the localization system.

The temporal consistency mechanism proved essential for stable detection during navigation. RViz visualization during an inspection mission with detected crack locations (red markers) overlaid on the SLAM-generated map is shown in Figure 16, demonstrating persistent spatial tracking of structural defects.

### 5.5. Static vs. Real-Time Performance Comparison

The performance gap between static dataset evaluation and real-time deployment across hardware configurations is quantified in Table 5. While static inference achieved 45 fps (U-Net) and 38 fps (two-stage), real-time operation decreased to 20 fps and 16 fps on the offboard GPU due to image acquisition, ROS messaging, temporal filtering, and visualization overhead. Onboard CPU-only processing further degraded to 5 fps and 2 fps, highlighting the importance of embedded GPU acceleration for practical deployment.

Despite lower throughput, real-time deployment demonstrated substantial qualitative improvements: the two-stage model detected thin/low-contrast cracks consistently missed by the baseline U-Net and produced fewer false positives, validating the refinement stage’s practical utility.

## 6. Discussion

This section discusses experimental results, highlighting performance gains and identifying limitations and opportunities for future work.

### 6.1. Contributions of the Two-Stage Architecture

The residual learning approach implemented through Pix2Pix refinement represents a novel contribution to automated crack detection, particularly in the context of real-time robotic systems. By training the second stage to predict corrections rather than complete segmentations, the method leverages the U-Net’s robust feature extraction while allocating additional model capacity to address specific failure modes. This architectural choice yields three primary benefits: (1) The refinement stage operates on compact residual representations rather than full RGB images, reducing memory bandwidth requirements and enabling higher throughput than would be achievable with a comparably sized single-stage model. (2) The adversarial training component of Pix2Pix encourages perceptually realistic crack boundary predictions, which empirical testing revealed to be more consistent with expert human annotations than the sometimes over-segmented boundaries produced by purely supervised training. (3) The two-stage decomposition facilitates modular system development. The U-Net baseline can be deployed alone when computational resources are limited, with the refinement stage activated when higher accuracy justifies the performance cost.

Quantitative analysis of error patterns revealed that the Pix2Pix refinement most significantly improved performance on three challenging crack subtypes: (1) Hairline cracks with widths below 2 mm, where the U-Net baseline frequently produced disconnected segments. (2) Cracks on highly textured surfaces, such as rough concrete, where background patterns generated false positives. (3) Cracks under non-uniform illumination, where shadows and highlights caused boundary localization errors. The improvements in these categories directly address the practical challenges encountered in real-world infrastructure inspection.

### 6.2. Real-Time Performance and Robustness

A notable difference was observed between model performance on the static dataset and during real-time robotic deployment (see Table 5). This disparity reflects the computational and communication overheads inherent in real-time robotic systems, where inference must operate within a full sensor–control loop rather than in an isolated image processing environment. The drop in frame rate also results from added operations such as depth filtering, temporal consistency checks, and RViz visualization, which are absent in static testing. These results emphasize the need for optimizing both model efficiency and system integration when transitioning from controlled datasets to onboard robotic environments.

Despite the lower frame rate, real-time deployment demonstrated substantial qualitative improvements. The two-stage model detected thin and low-contrast cracks that the baseline U-Net consistently missed and produced fewer false positives. However, some predictions exhibited increased discontinuity, particularly under motion blur or low illumination. These effects stem from frame-to-frame variation and the absence of temporal smoothing in the refinement stage. Future research could mitigate this through spatiotemporal architectures or recurrent refinement models to enhance continuity across frames.

Overall, although the real-time processing speed is lower, the improved detection robustness, lower false-positive rate, and successful onboard execution highlight the framework’s potential for practical autonomous inspection applications.

### 6.3. Limitations and Practical Considerations

Despite the promising results, several limitations constrain the current system’s applicability and real-time performance. While the CrackSeg9k dataset provided a strong foundation for training, domain shift issues emerged when deploying the model on diverse surface types encountered in real-world inspection scenarios. These challenges were partially mitigated through various preprocessing techniques, particularly image tiling, which helped improve model generalization across different surface textures and crack morphologies. However, this preprocessing adds complexity to the deployment pipeline and suggests that more robust domain adaptation strategies or expanded training datasets encompassing wider material varieties would be beneficial for future iterations.

Additionally, lighting variability also emerged as a limitation of the proposed system. Shadows and surface reflections observed during robot motion occasionally produced false-positive crack responses in the segmentation output. This effect was partially mitigated through the Pix2Pix adversarial training framework, where the discriminator learned to suppress texture-driven false positives, such as shadow edges, as well as through temporal filtering (Section 5.3.2), which removed short-lived detections during motion. While these strategies improved robustness under variable lighting, residual sensitivity to illumination changes remains. Future work should incorporate shadow-specific data augmentation, illumination normalization, and multi-spectral imaging to further enhance performance in outdoor and uneven lighting conditions.

While the system performs reliably in controlled indoor environments and generalizes reasonably well to campus images with moderate lighting variation, systematic outdoor validation in harsher construction environments remains an open challenge. Environmental factors requiring dedicated investigation include dust and airborne particulates, which may occlude camera optics or reduce surface contrast; uneven terrain, where vibrations may degrade perception quality despite previously demonstrated navigation robustness; and adverse weather conditions such as rain, fog, and temperature extremes, which fall outside the scope of the current evaluation. Although the demonstrated detection performance combined with established navigation capability provides a strong foundation for outdoor deployment, reliable operation in active construction zones will require additional environmental hardening and extensive field validation.

The most significant practical limitation, however, stems from the computational overhead of the detection pipeline (Table 3) when deployed on the onboard robotic computer, which lacks GPU acceleration. This constraint resulted in a processing rate of only 2 fps for the complete pipeline, fundamentally limiting the real-time capability of the system and preventing effective autonomous operation at typical inspection speeds. The necessity of performing crack detection on a separate offboard computer introduced additional operational complexity, as this external computing unit had to be physically transported alongside the robotic system, reducing mobility and deployment flexibility. This cumbersome setup highlights the critical need for more efficient computational architectures in future work.

To address these limitations, future research should pursue lightweight deep learning models specifically optimized for edge deployment, including the investigation of model compression techniques such as quantization, pruning, and knowledge distillation. Additionally, emerging generative AI algorithms that can achieve comparable detection performance with reduced computational requirements represent a promising avenue for enabling true real-time, onboard crack detection on resource-constrained robotic platforms.

### 6.4. Future Research Directions

Building upon the foundation established by this crack detection system, several key enhancements are planned to develop a comprehensive autonomous infrastructure inspection and repair platform.

- Robotic Manipulation Integration: The current system will be extended to incorporate the full functionality of the robotic arm currently attached to the platform. This integration will enable physical interaction with detected defects, transforming the system from a passive inspection tool into an active intervention platform. The robotic arm will be programmed to autonomously position repair tools at crack locations identified by the vision system, enabling precise, targeted maintenance operations.
- 3D Crack Profiling: To complement the current 2D vision-based detection, laser scanning profiling will be integrated to acquire in-depth information about detected cracks. This capability will enable quantitative assessment of crack severity through measurements of depth, width, and volumetric extent, which will be critical parameters for structural health evaluation and repair planning. The fusion of visual detection with depth data will provide a more complete characterization of surface defects and improve prioritization of repair interventions.
- Autonomous Crack Repair: The ultimate goal is to develop a fully autonomous crack repair system that can detect, assess, and remediate surface defects without human intervention. This will involve investigating suitable repair materials and application methods compatible with robotic deployment, such as automated sealant injection or epoxy application. The integration of detection, profiling, and repair capabilities will enable continuous, proactive infrastructure maintenance that can address defects before they propagate into major structural problems.

These enhancements will establish a truly autonomous inspection and repair system capable of real-time operation on resource-constrained robotic platforms.

## 7. Conclusions

This paper presents a two-stage real-time crack detection system integrated with a mobile robotic platform for autonomous infrastructure inspection. The system addresses critical gaps in existing approaches by combining deep learning perception with synchronized localization and autonomous navigation in a field-deployable package.

There are three distinct contributions to this research. First, a novel two-stage deep learning architecture coupling U-Net semantic segmentation with Pix2Pix residual refinement was developed, achieving 73.9 ± 0.6% mIoU and 76.4 ± 0.3% F1 score on the CrackSeg9k dataset while maintaining 16 fps real-time processing. The residual learning approach effectively enhanced boundary precision for thin cracks and suppressed texture-induced false positives. Second, a distributed ROS 2 Humble architecture integrating SLAM toolbox, AMCL localization, and Nav2 navigation enabled robust autonomous operation in GPS-denied environments with spatial localization precision below 5 cm. Third, comprehensive validation across simulation, controlled laboratory settings, and real-world deployment demonstrated the system’s practical viability, successfully detecting micro-cracks as small as 0.3 mm.

The system’s ability to identify both genuine structural defects and photorealistic crack decoys validates its robustness for field deployment. The temporal consistency mechanism, requiring detection persistence across 4 of 5 consecutive frames, proved essential for stable operation during navigation while maintaining sensitivity to genuine defects.

This technology can transform infrastructure inspection practices across multiple levels, such as facility managers, structural engineers, and regulatory inspectors. Currently, infrastructure inspection requires manual access to hazardous locations, extensive downtime, and suffers from inspector subjectivity and fatigue. With this autonomous system, facility managers can schedule continuous monitoring missions during off-peak hours, engineers can access georeferenced crack data for structural analysis, and regulators can verify inspection completeness through persistent digital records. The system’s integration with standard ROS 2 frameworks ensures compatibility with various robotic platforms, lowering adoption barriers for organizations transitioning from manual to automated inspection workflows.

Future development will integrate the onboard manipulator for active crack repair, incorporate 3D laser profiling for quantitative depth measurement, and implement lightweight deep learning architectures optimized for onboard edge deployment. These enhancements will enable fully autonomous detect-to-repair workflows, eliminating the need for offboard computing infrastructure and enabling deployment in remote or network-constrained environments such as bridges, tunnels, and post-disaster zones.

As infrastructure aging accelerates globally and inspection workforce shortages intensify, autonomous systems represent a critical pathway toward proactive, data-driven infrastructure management that can detect deterioration early, prioritize maintenance resources efficiently, and ultimately enhance public safety while reducing lifecycle costs.

## Figures and Tables

**Table 1:** Data Augmentation and Preprocessing Pipeline.

Category	Transformation	Parameters/ Probability (*p*)	Purpose/Effect
Geometric Transformations	Horizontal Flip	*p* = 0.5	Introduces left–right orientation variability
	Vertical Flip	*p* = 0.3	Adds top–bottom orientation variability
	Random 90° Rotation	*p* = 0.5	Ensures rotational invariance to crack orientation
	Shift Scale Rotate	Shift: ±10%, Scale: ±10%, Rotation: ±15°, *p* = 0.5	Simulates perspective and viewpoint changes during mobile robot navigation
Photometric Transformations	Random Brightness & Contrast	brightness_limit = 0.2, contrast_limit = 0.2, *p* = 0.3	Mimics illumination/exposure variations due to lighting and weather changes
Image Degradation Simulations	Gaussian Noise	Variance ∈ [10.0, 50.0], *p* = 0.2	Simulates sensor noise and compression artifacts
	Elastic Transformation	α = 1, σ = 50, *p* = 0.2	Introduces subtle local distortions resembling surface irregularities or lens aberrations
Validation & Testing Pre-processing	Resizing	384 × 384	Standardizes input image dimensions for inference
	Normalization	mean = [0.485, 0.456, 0.406], std = [0.229, 0.224, 0.225]	Normalizes using ImageNet statistics for numerical stability and transfer learning

**Table 2:** Comparative Performance of Crack Detection Methods.

Method	mIoU (%)	F1 Score (%)	FPS
YOLOv8-seg (n)	26.3	29.0	244
YOLOv11-seg (n)	29.5	36.4	200
U-Net + ViT [54]	71.8	-	-
HrSegNet-B16 [55]	78.4	-	182.0
HrSegNet-B48 [55]	80.3	-	140.3
CrackScopeNet [56]	82.1	-	-
U-Net (baseline)	70.2 ± 0.2	72.7 ± 0.5	45.2
Proposed Method (U-Net + Pix2Pix)	73.9 ± 0.6	76.4 ± 0.3	37.9

**Table 3:** Computational Cost of Residual Refinement.

Metric	U-Net Only	U-Net + Pix2Pix	Overhead
Parameters	13.4 M	42.6 M	+218%
FLOPs	69.9 G	109.1 G	+56%
Inference Time	22.1 ms	26.4 ms	+19%

**Table 4:** System-level performance metrics for real-time crack detection on the mobile robotic platform.

Metric	Value	Description
Processing Rate (U-Net only)	~20 fps	Baseline segmentation inference rate
Processing Rate (U-Net + Pix2Pix)	~16 fps	Two-stage pipeline inference rate
End-to-End Latency (U-Net)	50 ms	Frame acquisition to result publication
End-to-End Latency (U-Net + Pix2Pix)	62.5 ms	Complete two-stage processing delay
Temporal Filter Window	5 frames	Sliding-window size for consistency check
Temporal Detection Threshold	4/5 frames	Required persistence for confirmation
Minimum Crack Threshold	5.0%	Pixel percentage for frame-level detection
Operating Frame Resolution	384 × 384	Processed-image dimensions

**Table 5:** Performance comparison across configurations.

Configuration	Model	FPS	Latency (ms)
Static (inference only)	U-Net	45	22.2
	U-Net + Pix2Pix	38	26.3
Real-Time (offboard GPU)	U-Net	20	50.0
	U-Net + Pix2Pix	16	62.5
Real-Time (onboard CPU)	U-Net	5	200.0
	U-Net + Pix2Pix	2	500.0

## Data Availability

Data will be made available on request.

## References

[1] Farrar C.R., Keith W. (2012). Introduction. Structural Health Monitoring.

[2] Estes A.C., Frangopol D.M. (2003). Updating Bridge Reliability Based on Bridge Management Systems Visual Inspection Results. J. Bridge Eng..

[3] American Society of Civil Engineers (2025). 2025 Report Card for America’s Infrastructure.

[4] Koch C., Doycheva K., Kasi V., Akinci B., Fieguth P. (2015). A review on computer vision based defect detection and condition assessment of concrete and asphalt civil infrastructure. Adv. Eng. Inform..

[5] Jayaram M.A. (2023). Computer vision applications in construction material and structural health monitoring: A scoping review. Mater. Today Proc..

[6] Percepto (2023). Perception 2023: The State of Visual Inspection.

[7] U.S. Bureau of Labor Statistics (2024). Employment Projections—2023–2033. https://www.bls.gov/news.release/archives/ecopro_08292024.pdf.

[8] U.S. Bureau of Labor Statistics (2024). Occupational Outlook Handbook: Construction Laborers and Helpers. https://www.bls.gov/ooh/construction-and-extraction/construction-laborers-and-helpers.htm.

[9] Kaartinen E., Dunphy K., Sadhu A. (2022). LiDAR-Based Structural Health Monitoring: Applications in Civil Infrastructure Systems. Sensors.

[10] Yuan Q., Shi Y., Li M. (2024). A Review of Computer Vision-Based Crack Detection Methods in Civil Infrastructure: Progress and Challenges. Remote Sens..

[11] Ali L., Alnajjar F., Khan W., Serhani M.A., Al Jassmi H. (2022). Bibliometric Analysis and Review of Deep Learning-Based Crack Detection Literature Published between 2010 and 2022. Buildings.

[12] Weng X., Huang Y., Li Y., Yang H., Yu S. (2023). Unsupervised domain adaptation for crack detection. Autom. Constr..

[13] Wu Y., Li S., Li Y. (2026). Depth-aware RGB-D concrete crack segmentation and quantification using progressive cross-modal attention. Measurement.

[14] Musa A., Kakudi H., Hassan M., Hamada M., Umar U., Salisu M. (2025). Lightweight Deep Learning Models For Edge Devices—A Survey. Int. J. Comput. Inf. Syst. Ind. Manag. Appl..

[15] Halder S., Afsari K. (2023). Robots in Inspection and Monitoring of Buildings and Infrastructure: A Systematic Review. Appl. Sci..

[16] Huang M., Li X., Lei Y., Gu J. (2020). Structural damage identification based on modal frequency strain energy assurance criterion and flexibility using enhanced Moth-Flame optimization. Structures.

[17] Huang M., Ling Z., Sun C., Lei Y., Xiang C., Wan Z., Gu J. (2023). Two-stage damage identification for bridge bearings based on sailfish optimization and element relative modal strain energy. Struct. Eng. Mech. Int’l J..

[18] Huang M., Zhao W., Gu J., Lei Y. (2020). Damage identification of a steel frame based on integration of time series and neural network under varying temperatures. Adv. Civ. Eng..

[19] Kirthiga R., Elavenil S. (2023). A survey on crack detection in concrete surface using image processing and machine learning. J. Build. Pathol. Rehabil..

[20] Khan M.A.-M., Kee S.-H., Pathan A.-S.K., Nahid A.-A. (2023). Image Processing Techniques for Concrete Crack Detection: A Scientometrics Literature Review. Remote Sens..

[21] Lee D., Nie G.-Y., Ahmed A., Han K. (2022). Development of Automated Welding System for Construction: Focused on Robotic Arm Operation for Varying Weave Patterns. Int. J. High-Rise Build..

[22] Lee D., Nie G.-Y., Han K. (2023). Vision-based inspection of prefabricated components using camera poses: Addressing inherent limitations of image-based 3D reconstruction. J. Build. Eng..

[23] Hamishebahar Y., Guan H., So S., Jo J. (2022). A comprehensive review of deep learning-based crack detection approaches. Appl. Sci..

[24] Ronneberger O., Fischer P., Brox T., Navab N., Hornegger J., Wells W.M., Frangi A.F. (2015). U-Net: Convolutional Networks for Biomedical Image Segmentation. Proceedings of the Medical Image Computing and Computer-Assisted Intervention—MICCAI 2015.

[25] Liu Z., Cao Y., Wang Y., Wang W. (2019). Computer vision-based concrete crack detection using U-net fully convolutional networks. Autom. Constr..

[26] Mohammed M.A., Han Z., Li Y. (2021). Exploring the detection accuracy of concrete cracks using various CNN models. Adv. Mater. Sci. Eng..

[27] Cha Y., Choi W., Suh G., Mahmoudkhani S., Büyüköztürk O. (2018). Autonomous structural visual inspection using region-based deep learning for detecting multiple damage types. Comput. Aided Civ. Infrastruct. Eng..

[28] Redmon J., Farhadi A. (2018). Yolov3: An incremental improvement. arXiv.

[29] Yang H., Yang L., Wu T., Meng Z., Huang Y., Wang P.S.-P., Li P., Li X. (2022). Automatic detection of bridge surface crack using improved Yolov5s. Int. J. Pattern Recognit. Artif. Intell..

[30] Choi W., Cha Y.-J. (2019). SDDNet: Real-time crack segmentation. IEEE Trans. Ind. Electron..

[31] Panella F., Lipani A., Boehm J. (2022). Semantic segmentation of cracks: Data challenges and architecture. Autom. Constr..

[32] Lee D., Nie G.-Y., Han K. (2024). Automatic and Real-Time Joint Tracking and Three-Dimensional Scanning for a Construction Welding Robot. J. Constr. Eng. Manag..

[33] Badrinarayanan V., Kendall A., Cipolla R. (2017). Segnet: A deep convolutional encoder-decoder architecture for image segmentation. IEEE Trans. Pattern Anal. Mach. Intell..

[34] Chen L.-C., Papandreou G., Kokkinos I., Murphy K., Yuille A.L. (2018). DeepLab: Semantic Image Segmentation with Deep Convolutional Nets, Atrous Convolution, and Fully Connected CRFs. IEEE Trans. Pattern Anal. Mach. Intell..

[35] Bae H., Jang K., An Y.-K. (2021). Deep super resolution crack network (SrcNet) for improving computer vision–based automated crack detectability in in situ bridges. Struct. Health Monit..

[36] Amieghemen G.E., Ramezani M., Sherif M.M. (2025). Residual Pyramidal GAN (RP-GAN) for crack detection and prediction of crack growth in engineered cementitious composites. Measurement.

[37] Dai R., Wang R., Shu C., Li J., Wei Z. (2025). Crack Detection in Civil Infrastructure Using Autonomous Robotic Systems: A Synergistic Review of Platforms, Cognition, and Autonomous Action. Sensors.

[38] Yarovoi A., Cho Y.K. (2024). Review of simultaneous localization and mapping (SLAM) for construction robotics applications. Autom. Constr..

[39] Onatayo D., Onososen A., Oyediran A.O., Oyediran H., Arowoiya V., Onatayo E. (2024). Generative AI Applications in Architecture, Engineering, and Construction: Trends, Implications for Practice, Education & Imperatives for Upskilling—A Review. Architecture.

[40] Sampath V., Maurtua I., Aguilar Martín J.J., Iriondo A., Lluvia I., Aizpurua G. (2023). Intraclass Image Augmentation for Defect Detection Using Generative Adversarial Neural Networks. Sensors.

[41] Kim B., Natarajan Y., Preethaa K.S., Song S., An J., Mohan S. (2024). Real-time assessment of surface cracks in concrete structures using integrated deep neural networks with autonomous unmanned aerial vehicle. Eng. Appl. Artif. Intell..

[42] Delgado J.M.D., Oyedele L., Ajayi A., Akanbi L., Akinade O., Bilal M., Owolabi H. (2019). Robotics and automated systems in construction: Understanding industry-specific challenges for adoption. J. Build. Eng..

[43] Xiao B., Chen C., Yin X. (2022). Recent advancements of robotics in construction. Autom. Constr..

[44] Lee D., Han K. (2024). Vision-Based Construction Robot for Automated Welding in Real-Time: Proposing Fully Automated and Human-Robot Interaction. Autom. Constr..

[45] Lee D., Han K. (2025). Autonomous Navigation and Positioning of a Real-Time and Automated Mobile Robotic Welding System. J. Constr. Eng. Manag..

[46] Zhu H., Leighton B., Chen Y., Ke X., Liu S., Zhao L. (2019). Indoor navigation system using the fetch robot. Proceedings of the International Conference on Intelligent Robotics and Applications.

[47] Kulkarni S., Singh S., Balakrishnan D., Sharma S., Devunuri S., Korlapati S.C.R. (2022). CrackSeg9k: A Collection and Benchmark for Crack Segmentation Datasets and Frameworks. arXiv.

[48] Buslaev A., Iglovikov V.I., Khvedchenya E., Parinov A., Druzhinin M., Kalinin A.A. (2020). Albumentations: Fast and Flexible Image Augmentations. Information.

[49] Lee D. (2024). Real-Time Automated Mobile Robotic Crack Detection System_Gazebo Simulation. https://youtu.be/TNtV2WLi4f8.

[50] Lee D. Real-Time Automated Mobile Robotic Crack Detection System_Floor Experiment_Lab-Controlled. https://youtu.be/tSlldbQ8O78.

[51] Lee D. (2024). Real-Time Automated Mobile Robotic Crack Detection System_Wall Experiment_Lab-Controlled. https://youtu.be/2fDa-pLnY50.

[52] Lee D. (2024). Real-Time Automated Mobile Robotic Crack Detection System_2nd Floor in ERB at GSU. https://youtu.be/S_Qddbrxurk.

[53] Ogun E., Kim J., Lee D. (2025). Advanced Crack Detection in Building Structures Using Pix2Pix and U-Net Architectures. Proceedings of the Smart Materials, Adaptive Structures and Intelligent Systems.

[54] Noh J., Jang J., Jo J., Yang H. (2025). Crack Segmentation Using U-Net and Transformer Combined Model. Appl. Sci..

[55] Li Y., Ma R., Liu H., Cheng G. (2023). Hrsegnet: Real-time high-resolution neural network with semantic guidance for crack segmentation. arXiv.

[56] Zhang T., Qin L., Zou Q., Zhang L., Wang R., Zhang H. (2024). CrackScopeNet: A Lightweight Neural Network for Rapid Crack Detection on Resource-Constrained Drone Platforms. Drones.

[57] Yang X., Li H., Yu Y., Luo X., Huang T., Yang X. (2018). Automatic Pixel-Level Crack Detection and Measurement Using Fully Convolutional Network. Comput. Aided Civ. Infrastruct. Eng..

